# Crossing the Telemedicine Chasm: Have the U.S. Barriers to Widespread Adoption of Telemedicine Been Significantly Reduced? †

**DOI:** 10.3390/ijerph10126472

**Published:** 2013-11-28

**Authors:** Cynthia LeRouge, Monica J. Garfield

**Affiliations:** 1 Department of Health Management & Policy, College for Public Health and Social Justice, Saint Louis University, Salus Center, 3545 Lafayette Ave, Room 365, Saint Louis, MO 63104, USA; 2 CIS Department, Bentley University, 175 Forest Street, Waltham, MA 02452, USA; E-Mail: MGarfield@bentley.edu

**Keywords:** telemedicine, health reform, health technology barriers, health technology advancements, legal barriers

## Abstract

Barriers have challenged widespread telemedicine adoption by health care organizations for 40 years. These barriers have been technological, financial, and legal and have also involved business strategy and human resources. The article canvasses recent trends—events and activities in each of these areas as well as US health reform activities that might help to break down these barriers. The key to telemedicine success in the future is to view it as an integral part of health care services and not as a stand-alone project. Telemedicine must move from experimental and separate to integrated and equivalent to other health services within health care organizations. Furthermore, telemedicine serves as vital connective tissue for expanding health care organization networks.

## 1. Introduction

Telemedicine has the potential to play an integral role in providing medical information and services across space and time via telecommunication technologies ranging from the telephone to robotics [1]. One of the major goals of telemedicine is to enhance the delivery of health care to geographically disadvantaged and medically underserved populations, thereby providing an improved quality of care while decreasing costs [2]. Telemedicine also aligns with the shift in national focus from technology being used in isolation to technology being the means to both expand the reach of health care and to integrate health care services across patients and organizations. Modern telemedicine (closed circuit television) has been in existence for approximately 50 years, beginning in the late 1960s with projects such as those launched by the National Aeronautics and Space Administration (NASA) (with the U.S. Department of Public Health and Lockheed) and the Nebraska Psychology Institute (with Norfolk State Hospital) [3,4]. 

**Figure 1:**
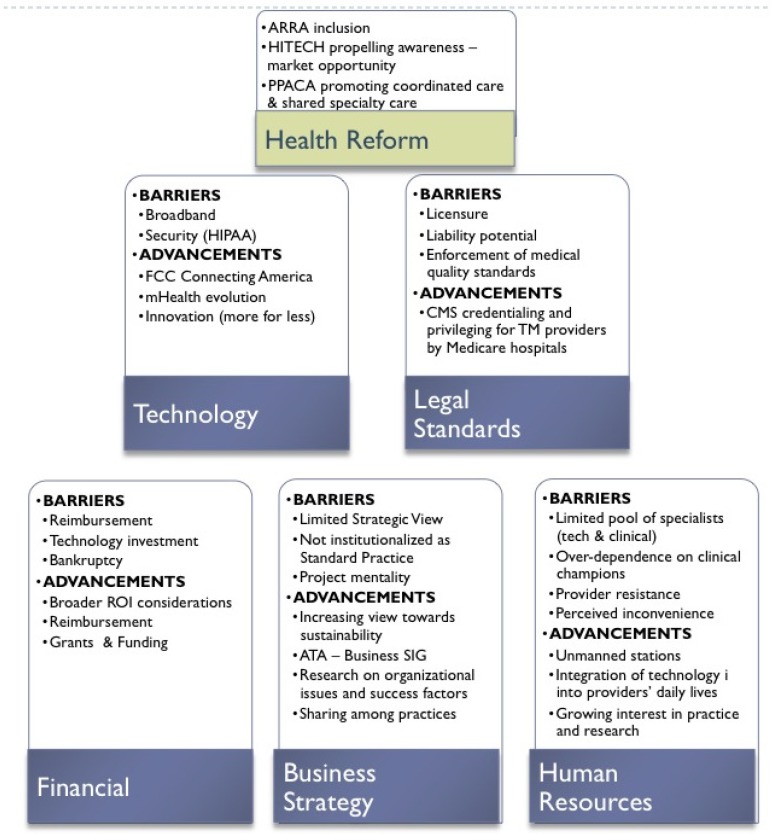
Telemedicine barriers and recent advancements.

Although telemedicine has a long history, its adoption has proven slow due to a multitude of barriers. These barriers have been technological, financial, and legal and have also involved business strategy and human resources. Recent events and activities in each of these areas in addition to U.S. health reform activities may do much to break down these barriers. These recent events and activities beget the question, “*Have the U.S. barriers to widespread adoption of telemedicine been significantly reduced?*” Figure 1 provides an outline of the key issues involved in answering this question. The remainder of the article will be a discussion of these issues, and we will conclude with insights on how to move forward.

## 2. Health Reform and Telemedicine

Telemedicine received a boost from the Health Reform Act’s focus on Health Information Technology (IT) as well as from funding aimed at improving quality, safety, efficiency, and reducing health disparities. The American Recovery and Reinvestment Act (ARRA) of 2009 included Health IT and telemedicine in its attempt to stimulate economic growth in specific businesses. The ARRA included $22 billion in government subsidies for the modernization of Health IT systems and $10 billion for health research and the construction of facilities (via the HITECH Act). 

“It is time to think boldly about the current health reform legislative environment and the unprecedented opportunities for not simply promoting the diffusion of telemedicine but, much more importantly, to establish telemedicine as an integral component of a more rational healthcare organization in this country [5].” Telemedicine has a key role to play in policy focused on health outcome priorities including reducing health disparities in access to health care, engaging patients and families in their personal health development, improving care coordination, and improving public health [6]. 

Telemedicine can be a pivotal force in working to achieve care coordination and improvements in health disparity outcomes to ensure that patients receive the proper care (based on clinical need and evidence-based medicine) at the appropriate site (closest to where they live and work, aided by electronic links) by a suitable provider (based on explicit and rational triage criteria) while avoiding duplication and waste (using uniform protocols for diagnostics and procedures) [5]. Connectivity and care coordination underlie the core elements of healthcare reform, namely, electronic health records (EHR), meaningful use, health information exchange (HIE), and accountable care organizations (ACO). The Accountable Care Organization (ACO) model was incentivized in §3022 of the Patient Protection and Affordable Care Act (PPACA). ACOs (which are closely connected with the Institute for Healthcare Improvement’s Triple Aim framework) are defined as an integrated “group of providers and suppliers who work together to coordinate care for the Medicare fee-for-service beneficiaries they serve” [7]. The Centers for Medicare and Medicaid Services (CMS) has identified a three-part goal for ACOs: (1) better care for individuals; (2) better health for populations; and (3) lower growth in expenditures. CMS, however, has not defined specific “means” for achieving these three goals, although many structural and organizational changes will likely be needed. The American Telemedicine Association (ATA) has suggested the beneficial uses of telemedicine in the ACO model [8]. The underlying premise is that shared specialty services, coordinated care with more service sites, and easier access can reduce the cost of care. Numerous organizations, including the ATA, have called for the repeal of certain restrictions on telemedicine for Medicare reimbursement to work toward the goals of the ACO model [8]. 

Furthermore, telemedicine is increasingly being seen as a means to facilitate the Patient Centered Medical Home (PCMH) model [9,10]. The Agency for Healthcare Research and Quality has specified telemedicine as a Health IT application that can facilitate the medical home principles of:(a) patient-centered, whole person orientation; (b) comprehensive, team-based care; (c) coordinated care; (d) continuous access to care; and (e) a systems-based approach to quality and safety [11]. As part of this movement, growing numbers of federal and state agencies are providing programs and grants to telemedicine services as a means to engage patients and families in their personal health development through the PCMH concept [12,13,14]. It is in recognition of the aforementioned principles and other considerations that a number of thought leaders take the position that telemedicine is essential to health care reform [9,15]. 

### 2.1. Technology

#### 2.1.1. Barriers

The lack of broadband infrastructure has proven challenging for the advancement of many forms of telemedicine, specifically high demand video and store-and-forward services, which require expansive health networks [16]. The broadband penetration rate in the U.S. (26.4 connections per 100 inhabitants) makes it 15th in the ranking of countries by the Organization for Economic Cooperation and Development (OECD) [17], down from its ranking of 6th in 2002. Fifteen percent of American adults (over 18 years old) do not use the Internet as of May 2013, which may make the application of widespread home telemedicine difficult to implement [18].

As the integration and connectivity between health care entities increase, the importance of a robust technology security policy to protect confidential patient financial and medical records also increases [16]. Security needs include data confidentiality and integrity (during both transmission and retention) while enhancing availability and ease of use, as well as a need to define the standards for minimum requirements. A recent systematic review of the literature related to telemedicine security indicated that there is a dearth of standardization in telemedicine security [11]. 

#### 2.1.2. Recent Advancements

On 17 March 2010, President Obama proposed “Connecting America: The National Broadband Plan” to assist in the proliferation and improvement of broadband networks across the United States [19]. This plan calls for the build-out and improvement of medical networks that facilitate remote patient monitoring, electronic health records, and other technology-based health services such as telemedicine [11]. Actualizing the plan to help develop dedicated health bandwidth across an increasing number of distributed health networks (mobile and land based) will facilitate the expansion of video consultation, remote patient monitoring, and connected-care solutions at a significant cost savings [19]. As of February 2013, it appears that the U.S. is on the path to greater broadband connection; the U.S. is leading the world in the adoption of 4G/LTE mobile broadband and has the second lowest cost for entry pricing in Organization for Economic Co-operation and Development (OECD) countries [20].

Furthermore, the evolution of technologies is accommodating more extensive capabilities using less bandwidth. Cloud computing continues to grow with the utilization of widespread relatively high-speed mobile devices (e.g., iPads, iPhones, Android devices, or other smartphones). These devices support the development of mobile health concepts (mHealth) in becoming a viable avenue that can influence telemedicine possibilities. This method of connectivity provides a low cost and easy-to-use method for health user interaction, as these devices are becoming ubiquitous and embedded into the daily routines of many patients [21,22]. Conceptual privacy frameworks are also starting to emerge for mHealth [23].

Technological innovation also includes the development of new technologies such as handheld telemedicine kits, biosensor recliner chairs, telemedicine robots, and sensors to detect a person falling [24]. As an indirect form of teleconsultation, research is underway to investigate the role that computer avatars may play in enhancing care for conditions such as alcoholism [25]. These innovative efforts expand the possibilities for and also potentially the ease of entry to providing more useful telemedicine services. 

### 2.2. Legal

#### 2.2.1. Barriers

Historically, the challenge of medical licensure or “credentialing” for multi-state service provision by medical providers has been burdensome and has therefore restricted growth across state lines [26]. Even when licensing is in place, it is often difficult to work within multiple different health organizations because of privileging procedures within the organizations. Furthermore, the legalities surrounding virtual medical services can sometimes be inconsistent, vague, and increase liability concerns [27]. Quality standards and protocols also lack uniformity, which makes it difficult to develop a framework within which health organizations may operate [28]. Medical malpractice and liability issues continue to be areas where the law is unclear in terms of telemedicine practices [29,30], leaving hospitals and doctors open to unknown legal obligations and responsibilities. With the majority of health care regulations being governed at the state level, these barriers continue to plague the use of telemedicine [30]. For instance, the Delaware General Assembly Title 24 Professional Regulation, Section 9.2.1.4, states that: “licensees shall not evaluate or treat a client with speech, language, or hearing disorders solely by correspondence. Correspondence includes telecommunication” [31]. 

#### 2.2.2. Recent Advancements

In June of 2010, the Center for Medicare and Medicaid Services (CMS) proposed nationwide credentialing and privileging for health care professionals [32]. CMS issued a final rule on 5 May 2011 (effective 2 July 2011), offering hospitals and critical access hospitals (CAHs) the option to streamline the credentialing and privileging processes for physicians and non-physician practitioners providing telemedicine services through the use of a uniform application and expedited license model. The *Code of Federal Regulations* (CFR) 42 CFR 485.616 was modified in October 2012 to enable these credentialing and privileging decisions to be made by the distant-site hospital when telemedicine services are provided to a CAH. While licensing is typically a state-level issue, ten state boards issue special purpose licenses, telemedicine licenses or certificates, or licenses to practice medicine across state lines to allow the practice of telemedicine, and fifteen states currently require private insurance companies to cover telemedicine services to the same extent as face-to-face consultations [33]. Two additional bills have been referred to committee in 2013: HR 3077, which would permit certain Medicare providers licensed in a state to provide telemedicine services to certain Medicare beneficiaries in a different state, and HR 2001, which will enable VA doctors to practice their health profession in any state if the healthcare professional is using telemedicine to provide treatment. Congressman Gregg Harper is also working on a bill that would significantly expand the role of telemedicine within Medicare and Medicaid. 

### 2.3. Financial

#### 2.3.1. Barriers

The clearly discernible economic benefits of telemedicine may favor the patient through the reduction of patient costs by reducing the travel time, decreasing patient waiting time, decreasing patient anxiety, and minimizing time out of work [34]. The return on investment (ROI) from the perspective of the health care organization is unclear. There are some contexts where a favorable ROI proposition for telemedicine seems to be readily apparent. In a home health context, provider travel costs are greatly reduced with the use of telemedicine, and it appears that home health agencies recognize these savings. A 2007 study reported that approximately 21% of all U.S. home health and hospice agencies were using some form of telemedicine [35]. 

However, the ROI is not as readily apparent for many other services. Telemedicine is plagued by hazy economic cost and revenue measures, which are complicated by insurance reimbursement challenges. Specifically, health insurance providers traditionally only reimburse for services that mimic the normal interactions between patients and health institutions [16]. Moreover, the reimbursement situation is somewhat recursive in that additional financial data are needed to provide evidence to insurance companies that telemedicine provides a financial benefit in reducing the higher costs of increased medical care [36]. The future of reimbursement further complicates the financial landscape as specific reimbursement structures vary from state to state. 

Economic benefits (particularly when reimbursement is questionable) may not justify the equipment and communication investment costs incurred to install and maintain selected telemedicine services. Furthermore, recent economic conditions fueling the bankruptcy and closure of many health facilities in rural communities (often telemedicine spoke sites) may stunt telemedicine expansion to communities needing specialized services [16].

#### 2.3.2. Recent Advancements

There is growing evidence indicating that the benefits of telemedicine exceed the costs and that these benefits accrue to providers, patients, and society at large [9]. In looking for financial benefits to the health care organization, some organizations are recognizing downstream revenues and well as the ability to avoid penalties (*i.e.*, readmission penalties) facilitated by telemedicine services in their ROI assessments. Opportunities may also exist to leverage telemedicine to achieve recognitions (such as centers of excellence) or the authorization to provide a particular form of care at under-sourced sites in a health care network to capture business that would otherwise go elsewhere. 

In addition, there is some movement in reimbursement rules. Both Medicare and Medicaid have announced that they will be expanding telemedicine coverage (which may incentivize ACO development). The current federal Medicaid statute no longer recognizes telemedicine as a distinct service and has made a variety of Healthcare Common Procedure Coding System (HCPCS) codes (e.g., T1014 and Q3014) and Current Procedural Terminology (CPT) codes and modifiers (GT, U1-UD) available to those states that choose to cover such services under their Medicaid coverage [37]. As of 2013, Medicare pays for Medicare Part B services that are furnished via real time interactive video including patient health education, telehealth consultations, and some mental health services [38]. CMS is also working to extend telemedicine coverage through additional reimbursable services (e.g., transitional care management for post-discharge) and revisiting their urban/rural definitions (CMS has historically used strict county-based classifications to enforce its rural-only rule for telemedicine coverage) [39]. In addition, from the general payer front, on 2 March 2010, Virginia’s legislature unanimously approved a bill that would require private health insurers, health subscription plans, and HMOs to cover the costs of health care services provided through telemedicine [40]. As of July 2013, eighteen other states have also enacted legislation requiring private sector insurance companies to pay for telemedicine services. While all of these states mandate coverage, not all require reimbursement rates on par with rates for face-to-face services. There is promise that other states will follow these leaders. 

With regard to finances, US government spending for telemedicine can help defray the costs via grants for demonstrations and research, direct telemedicine services by federal agencies for covered populations, and reimbursement for remote medical services under Medicare [28]. Furthermore, when local and regional health care decision makers include telemedicine services in the mix of included services, managed care initiatives such as ACOs and PCMHs can change the way we pay for telemedicine services and ultimately impact their feasibility [41]. 

### 2.4. Business Strategy

#### 2.4.1. Barriers

To sustain telemedicine as a service, a strategic vision and a supportive organizational context are required. A supportive organizational context includes: (a) the provision of an overarching architecture and infrastructure [42]; (b) strong program management [42]; (c) thorough needs analysis and detailing applications to match the identified needs [42]; (d) suitable technology, technical, and operational support available at both the hub and spoke sites for the proposed application [16]; (e) adequate training and orientation of providers in the effective use of telemedicine technologies [16]; and (f) marketing services to promote the use of the telemedicine service both internally and externally. Without sufficient organizational support, telemedicine often fails to become institutionalized and a part of standard practice. Instead, a telemedicine effort is often viewed by many health organizations as an adjunct project rather than as a sustainable service that can provide ongoing improvements in patient care and medical protocols [43]. Demonstrating that telemedicine can be financially sustainable is perhaps the most difficult organizational issue resulting from challenging reimbursement situations and the challenges of gathering proper metrics, calculating the associated costs, and collecting revenue data [43]. One of the major business challenges for telemedicine is to integrate it into the existing payment models. These models include traditional fee for service, capitated/negotiated rates, employee wellness/prevention incentives, pre-tax spending accounts, and over-the-counter self-pay [44].

#### 2.4.2. Recent Advancements

Telemedicine is still considered a strategic gray area for most hospitals and institutions. However, sustainable business models are evolving through practice sharing avenues and research. Efforts by the ATA (particularly the Business and Finance Special Interest Group [41]) and Office for the Advancement of Telehealth (OAT, including efforts by the OAT funded Telehealth Resource Centers [45]) facilitate sharing guidelines and lessons learned among telehealth programs to facilitate sustainability and a strategic perspective. Organizational and workflow issues are an active and growing part of interdisciplinary research [46]. In the telemedicine domain, such studies promote better understanding of the key relationships and factors for sustainable success [25,47]. 

### 2.5. Human Resources

#### 2.5.1. Barriers

A successful telemedicine implementation requires not only access to a specialty providers’ knowledge, but these providers must also have the skills and willingness to operate the required medical telecommunication tools that facilitate the telemedicine process. Thus, another challenge is the inadequate pool of specialty providers available to meet the initial needs and growing stages of telemedicine [16]. When specialty provider shortages exist, many of the responsibilities of providing telemedicine service fall upon a limited number of physician champions. These clinical champions of telemedicine services may find that they are subject to a high level of on-call availability and scheduling challenges, particularly in cases of rapid telemedicine adoption from spoke sites. Such a situation may detract from further provider participation (*i.e.*, fewer champions emerging or existing champions not promoting expansion and future telemedicine efforts) and may lead to champion turnover. 

Aside from the issue of the shortage of telemedicine specialty providers, researchers have noted resistance from providers to telemedicine technologies due to a lack of equipment training, fear of patient loss, medical liability [48], and the fact that using telemedicine is often inconvenient for the medical provider (*i.e.*, if the telemedicine equipment requires that they use it in a designated room that takes them outside of their standard work path).

#### 2.5.2. Recent Advancements

Technological innovations may help address some of the human resource challenges. For example, the US military is investigating various promising avenues of providing health services via robotic telesurgery [49]. In addition, there has been a spread of walk-in, “self-service” telemedicine kiosks (e.g., Healthspot stations) containing high-definition videoconferencing systems with medical peripheral devices, where patients can connect with remotely located medical providers to treat more common and minor health needs [24]. 

The increasing use of electronic health records and other health IT is embedding technology use into mainstream medical practice (not to mention the daily use of personal technology gadgets). As a result, an ever-increasing number of providers are becoming more technologically savvy and recognizing that many of their patients are also technologically savvy. Research indicates that patients who have used telemedicine services are generally satisfied with the telemedicine experience they receive (over a 90% satisfaction rate) [50,51]. This could encourage patient demand. A growing aging population potentially open to telemedicine, coupled with a shortage of health care providers, may become some of the leading drivers of telemedicine adoption. As these providers recognize that their patients are satisfied with telemedicine services, it may diminish providers’ resistance to telemedicine.

Finally, interest in telemedicine among the practitioner and research community appears to be rising. The ATA has noted an increasing level of membership and participation [52] and accredited telemedicine training programs now exist [53]. Logic would indicate that this increased participation in the telemedicine community and public awareness could spawn additional interest from providers to participate in telemedicine efforts, yielding visionary champions. Some specialized providers championing telemedicine are banding together to form private virtual medicine firms. When resident providers are not available to fill the needs and test possibilities, up-and-coming private firms of virtual medical specialists allow health care organizations to outsource specialized care using remote clinical consultations [41]. 

## 3. Conclusions—Road to the Future

Throughout the past sixty years, telemedicine has grown from its infancy to a noticeable and growing force in today’s health landscape [54]. Many barriers to the widespread adoption of telemedicine are crumbling, paving a path for the widespread adoption of telemedicine. The recent advancements discussed above enable the health care majority to adopt telemedicine and can push the use of telemedicine to the next level on the adoption curve. However, the stakeholders involved will be the final determining factor on whether telemedicine makes it to the next level on the adoption curve. The remaining human, policy, and organizational issues surrounding telemedicine need attention from cross-functional research, practice, and policy teams to promote understanding and action to overcome remaining barriers that challenge the proliferation of these life-saving services and networks. Telemedicine plays a role in the future strategy of the healthcare industry and should be at the forefront of Health IT research. Modern concepts of telemedicine expansion include not only medical specialty and home health care delivery service lines but also applying telemedicine to the contexts of disease management, clinical decision support systems, and disaster preparedness and response [55].

We believe that the key to telemedicine success in the future is to view it as an integral part of health care services and not as a stand-alone project. It must move from experimental and separate to integrated and equivalent to other health services within health care organizations. Moreover, this vehicle of care must take center stage in strategic discussions of holistic health care systems. The integration of medical information systems (e.g., telemedicine and electronic health records) between and within health organizations provides the connective tissue for holistic health care. This connective tissue is necessary and vital to expanding health care organization networks and the potential emergence of “Big Med” with a focus on “delivering a range of services to millions of people at a reasonable cost and with a consistent level of quality” [56]. Virtual medical centers which serve multiple states and telehealth networks that deliver services from any point on the network to any other point (as opposed to a central hub delivering services to a spoke site) are the incipient telemedicine substance of this connective tissue [41].

To meet the current demands of health care policies and business models that focus on health networks, cost reduction, and improved health outcomes, telemedicine must be part of the health care portfolio to bring quality treatment to patients despite physical location. With the U.S. barriers that have inhibited the widespread adoption of telemedicine crumbling, we are at a pivotal point in telemedicine use and adoption where tactical deployment can lead to better health care, lower costs, and more equity in healthcare delivery in the U.S.
